# Anti-Inflammatory Response and Muscarinic Cholinergic Regulation during the Laxative Effect of *Asparagus cochinchinensis* in Loperamide-Induced Constipation of SD Rats

**DOI:** 10.3390/ijms20040946

**Published:** 2019-02-21

**Authors:** Ji Eun Kim, Ji Won Park, Mi Ju Kang, Hyeon Jun Choi, Su Ji Bae, You Sang Choi, Young Ju Lee, Hee Seob Lee, Jin Tae Hong, Dae Youn Hwang

**Affiliations:** 1 Department of Biomaterial Science, College of Natural Resources and Life Science/Life and Industry Convergence Research Institute, Pusan National University, Miryang 627-706, Korea; prettyjiunx@naver.com (J.E.K.); pjw08260824@naver.com (J.W.P.); beautifulbead@naver.com (M.J.K.); rudwns546@naver.com (H.J.C.); suji130501@naver.com (S.J.B.); choiyusang@gmail.com (Y.S.C.); youngju0831@naver.com (Y.J.L.); 2 College of Human Ecology, Pusan National University, Busan 609-735, Korea; heeseoblee@pusan.ac.kr; 3 College of Pharmacy, Chungbuk National University, Chungju 361-763, Korea; jinthong@chungbuk.ac.kr

**Keywords:** constipation, *Asparagus cochinchinensis*, inflammation, cytokines, acetylcholine esterase, muscarinic acetylcholine receptors

## Abstract

Several types of saponins and herbal plants containing saponins have been reported to have anti-inflammatory or laxative activities. To verify the therapeutic effects of saponin-enriched extracts of *Asparagus cochinchinensis* (SPA) on the anti-inflammatory response and on the cholinergic regulation in the gastrointestinal system, an alteration on the constipation phenotypes, on the inflammatory responses, and on the muscarinic cholinergic regulation were investigated in the transverse colons of Sprague Dawley (SD) rats with loperamide (Lop)-induced constipation after the treatment of SPA. Significant increases were observed on the total number of stools, the gastrointestinal transit, the thickness of the mucosal layer, the flat luminal surface, the number of paneth cells, and the lipid droplets in the Lop + SPA-treated group as compared to the Lop + Vehicle-treated group. SPA treatment induced the recovery of inflammatory cytokines (TNF-α, IL-1β) and IL-6), inflammatory mediators (NF-κB and iNOS), the total number of infiltered mast cells, and mucin secretion. Also, some similar improvements were observed on the levels of acetylcholine esterase (AChE) activity and on the phosphorylation of myosin light chains (MLC) as well as the expression of muscarinic acetylcholine receptors M2/M3 (mAChR M2/M3) and their mediators. The results presented herein provide the first strong evidence that SPA stimulates anti-inflammatory responses and the muscarinic cholinergic regulation when exerting its laxative effects in the chronic constipation of Lop-induced models.

## 1. Introduction

Constipation is an acute or chronic gastrointestinal disease characterized by infrequent bowel movements, difficulty during defecation, incomplete bowel evacuation, and hard and dry feces [1]. Although there are numerous treatments for this disease, constipation is best treated by making simple diet changes by incorporating more fiber into the diet, by drinking plenty of fluids, and by adding exercise in the patient’s daily routine. Chemical drugs (laxatives) such as senna, correctol, exlax, senokot, and gaviscon are frequently administered to patients with constipation [2]. These drugs act as stimulants to increase the bulkiness and to soften stool or as osmotic agents that enhance water flow into the colon to promote elimination and to trigger bowel movements. However, most laxatives have accompanying undesirable side effects, including artery contraction, coronary spasms, and myocardial infarction [3,4,5]. Hence, the primary focus for constipation therapy remains on the identification of novel laxatives derived from natural products that have no side effects.

Since very few studies have investigated the inflammatory responses and cholinergic regulation during the laxative effects of natural products, the subjects remain relatively unknown. Atractylodin, a widely used oriental medicine, decreases the expression of proinflammatory cytokines (TNF-α, IL-1β, and IL-6) and inflammatory mediators (iNOS and NF-κB) as well as reduces the number and moisture content of stools in constipation-prominent models [6]. Also, the downstream signaling pathway of some mAChRs belonging to cholinergic regulation significantly recovered in the Lop-induced constipation models after the treatment with extracts from *Liriope platyphylla* [5], Galla Rhois [7], or red *L. platyphylla* (RLP) [8]. However, these previous studies did not analyze the anti-inflammatory effects and cholinergic regulation concurrently, although these two responses need to be considered as key factors of chronic constipation.

To date, there are few reports of major active ingredients, although many studies have reported the laxative effects of various herbal plants. Genkwanin-5-O-beta primeveroside collected from agarwood was first identified as the main constituent contributing to laxative effects. When exposed to 1000 mg/kg of the compound, the frequency and weight of stools in male ddY mice were significantly increased [9]. Based on the results of previous studies, tannin and saponin have recently been considered potential laxative candidates [10,11]. Tannin is distributed in several plant extracts that exhibit laxative activities, based on their ability to increase the number and weight of feces. Tannin is also detected in the leaf aqueous extract of *Mareya micrantha* Mull. Arg. [10]; the methanol extract of *Senna macranthera* [11]; the aqueous extracts of *Aloe ferox* Mill. [12]; the aqueous-methanol extracts of *Fumaria parviflora* [13], *Urginea indica* Kunth. [14], *Phyllanthus emblica* [13], and gallotanin-enriched galla rhois (GEGR) [7]. Herbal plants containing saponin also exhibit laxative properties including the ability to increase intestinal motility, the frequency and weight of stools, and the ileum tension. Such compounds include extracts of *A. ferox* Mill. [12], *Ficus carica* paste [15], and the aqueous extract of *L. platyphylla* (AEtLP) [5]. Furthermore, saponin as well as extracts containing saponin inhibit the pro-inflammatory responses in cecal ligation, puncture (CLP)-induced sepsis mice, and dextran sulfate sodium (DSS)-induced mouse colitis models [16,17,18]. Ginsenoside Re exerts cholinergic stimulation and inhibitory effects on the contractility of isolated jejunal segments [19,20]. The laxative effects, inflammatory response, and cholinergic regulation of saponin-enriched extracts of *Asparagus cochinchinensis* (SPA) in the Lop-induced constipation model have not been fully investigated, although seven steroidal saponins were detected in the roots of *A. cochinchinensis* [21,22].

To evaluate the possibility of developing a new natural medicine, the present study was undertaken to investigate the inflammatory responses and cholinergic regulation during the laxative activity of SPA in a Lop-induced constipation model. Our results provide the first scientific evidence that SPA is a saponin-containing natural product that successfully induces laxative effects in the constipated animal model through the suppression of the inflammatory response and the recovery of the muscarinic cholinergic regulation.

## 2. Results

### 2.1. Effect of SPA Administration on the Feeding Behavior and Excretion Parameters

**Figure 1:**
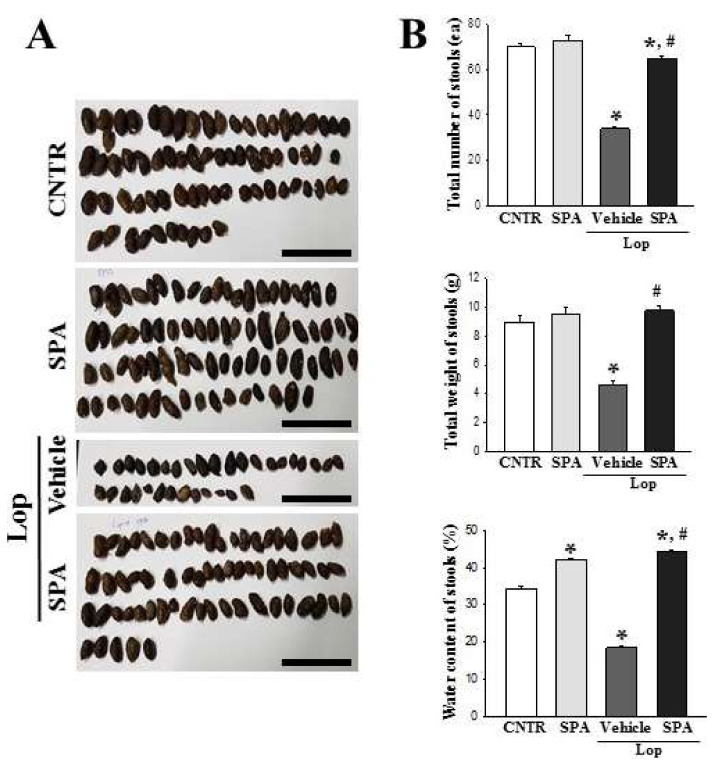

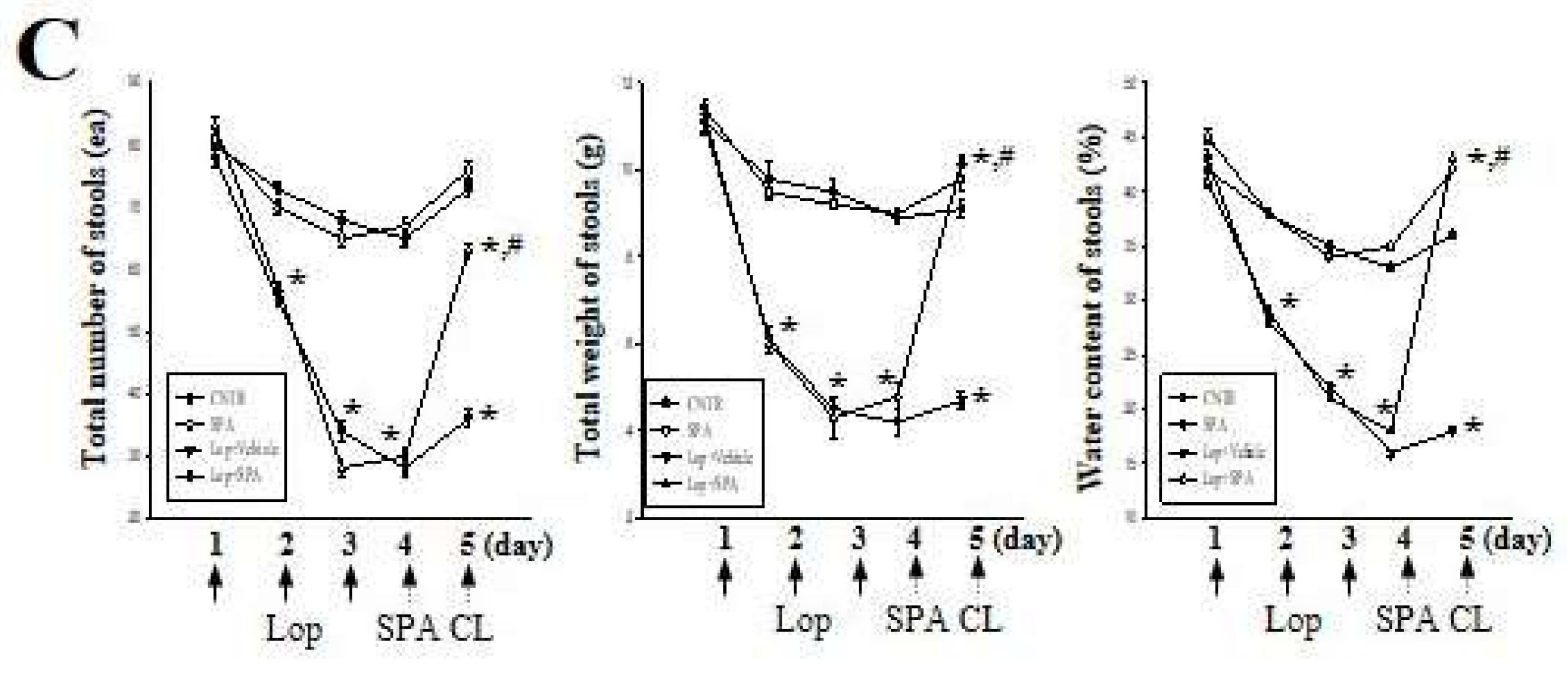
The number, weight, and water content of the stools of Lop-induced constipated SD rats after SPA administration. (**A**) The digital camera images of the stools were taken immediately after collection from the metabolic cage. Scale bar was represented 3 cm. (**B**) At 5 days, the total number and weight of stools were measured as described in the materials and methods. The stool water content was calculated using the weight of fresh stools and the dried weight. (**C**) From the 1st day to the 5th day, the total number, weight, and water content of stools was measured as described in the materials and methods. Six to seven rats per group were used in the stools’ collection, and each parameter was assayed in triplicate. The data are reported as the mean ± SD. * *p* < 0.05 compared to the CNTR group. # *p* < 0.05 compared to the Lop + Vehicle-treated group. CL, final collection.

To investigate whether SPA exposure affects the feeding behavior and the excretion of constipated Sprague Dawley (SD) rats, we assessed alterations in the food intake; water consumption; and the number, weight and water contents of stools in Lop-induced constipated SD rats after a single administration of SPA. As shown in Table 1, there are no significant alterations in the body weight, food intake, or water consumption. However, decreases observed in the stool number, weight, and water content as well as the round form of hard stools of the Lop + Vehicle-treated group were almost recovered in the Lop + SPA-treated group relative to the non-constipation (CNTR) and SPA-treated groups (Figure 1). However, a reverse pattern of the stool parameters was observed for urine volume, and the enhanced urine volume after Lop treatment decreased after the administration of SPA (Table 1). These results indicate that SPA administration stimulates the excretion of Lop-induced constipation in SD rats without a significant alteration in their feeding behaviors.

### 2.2. Effect of SPA Administration Gastrointestinal Motility

**Figure 2:**
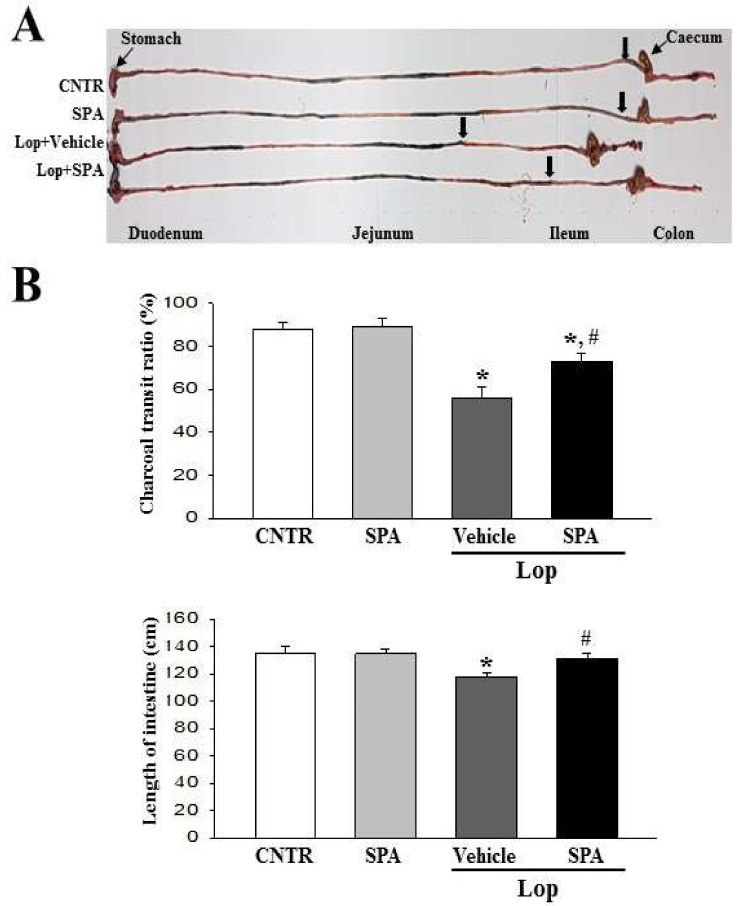
The gastrointestinal transit ratio and the intestine length: (**A**) An actual image of the charcoal meal transit and the intestine. The total intestinal tract was excised from a rat of the subset groups treated with charcoal meal powder. Their morphology was observed using a digital camera. The arrows indicate the position of the charcoal meal. (**B**) The transit ratio of the charcoal meal and the length of intestine: The total distance travelled by the charcoal meal from the pylorus was measured. The charcoal meal transit ratio was then calculated using the total length of the intestine and the distance of the charcoal meal. Six to seven rats per group were used in the gastrointestinal transit ratio test, and the charcoal meal transit distance and intestine length were measured in triplicate. The data are reported as the mean ± SD. * *p* < 0.05 compared to the CNTR group. # *p* < 0.05 compared to the Lop + Vehicle-treated group.

To investigate whether the SPA administration can induce changes in the gastrointestinal motility, the charcoal meal transit test was performed in Lop + SPA-treated SD rats. The propulsion of the charcoal meal was significantly decreased 37.5% in the Lop + Vehicle-treated group compared with CNTR group. However, this propulsion was recovered after the administration of SPA (Figure 2). Also, a similar pattern was observed on the length of the intestine. These results suggest that the SPA administration can promote gastrointestinal motility in the Lop-induced constipation model.

### 2.3. Effect of SPA Administration on Histological Structure of the Colon

**Figure 3:**
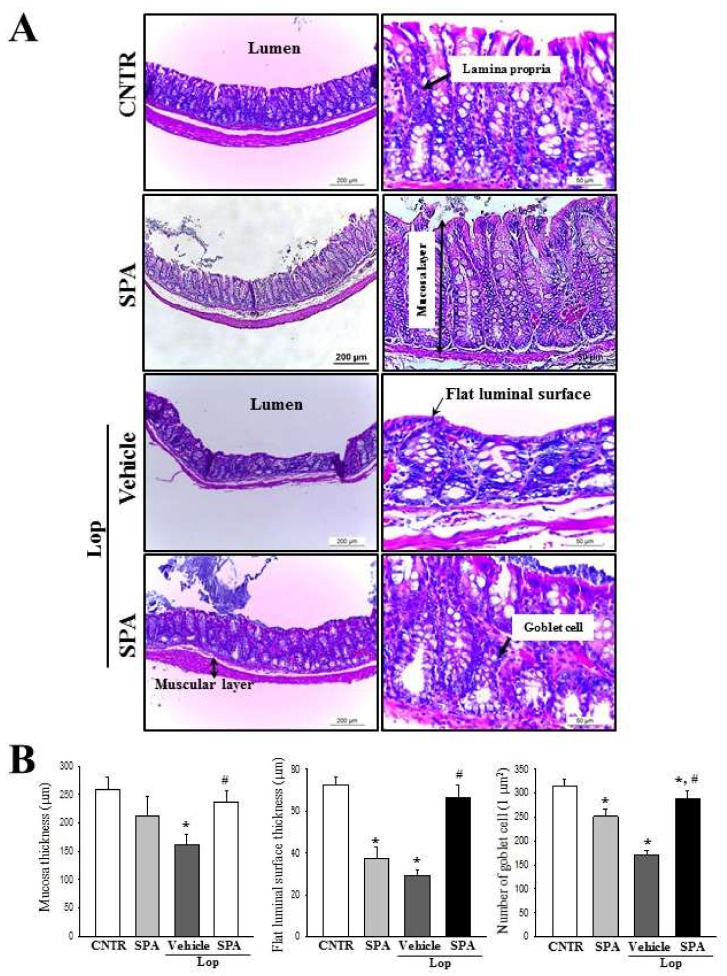
The histological structures of the colons in Lop-induced constipated rats after SPA administration: (**A**) The hematoxylin and eosin (H&E)-stained sections of the colon from the CNTR, SPA-, Lop + Vehicle- or Lop + SPA-treated group were observed at 100× (left column) and 200× (left column) using a light microscope.(**B**) The histopathological parameters were determined using the Leica Application Suite (Leica Microsystems). Five to six rats per group were used in the histological analysis, and each parameter was measured in duplicate in two different slides. The data are reported as the mean ± SD. * indicates *p* < 0.05 compared to the CNTR group. # indicates *p* < 0.05 compared to the Lop + Vehicle-treated group.

Alterations in the histological structure of the Lop-induced constipated colon were investigated after SPA administration. To accomplish this, alterations in the histological parameters associated with laxative effects were measured in the hematoxylin and eosin (H&E)-stained colons of the subset groups. The SPA-alone-treated group showed a structure similar to the CNTR group. A significant decrease in the thickness of mucosa and flat luminal surface was observed in the Lop + Vehicle-treated group relative to the CNTR group and the SPA-treated group. However, a dramatic increase of these parameters, 2.83 folds and 39%, respectively, was observed following the Lop + SPA cotreatment when compared with the Lop + Vehicle-treated group (Figure 3). Furthermore, the number of goblet cells was 41% lower in the Lop + Vehicle-treated group than in the CNTR group. However, although a recovery in the number of goblet cells was observed in the Lop + SPA-treated groups, the levels did not reach those of the CNTR group (Figure 3). Taken together, these findings show that SPA administration contributes to the recovery of abnormalities observed in the colon of Lop-induced constipated SD rats.

**Figure 4:**
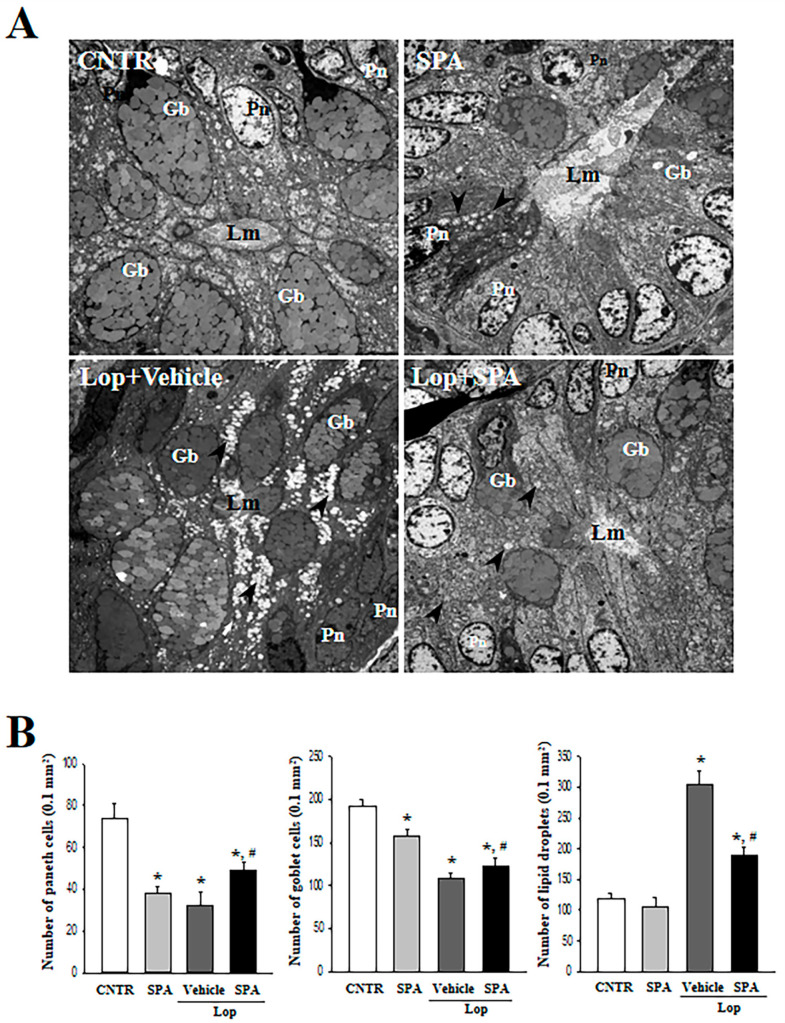
An ultrastructure image of the colon after SPA administration: (**A**) The ultrastructure of the crypt in the CNTR, SPA-, Lop + Vehicle- and Lop + SPA-treated groups were viewed by TEM at 4000× magnification. (**B**) The number of paneth cells, lipid droplets, and goblet cells were measured using Leica Application Suite (Leica Microsystems, Switzerland). The arrow indicates a lipid droplet distributed around the lumen of the crypt. Two to three rats per group were used in the TEM analysis, and each parameter was measured in duplicate in two different slides. The data are reported as the mean ± SD. * indicates *p* < 0.05 compared to the CNTR group. # indicates *p* < 0.05 compared to the Lop + Vehicle-treated group. Lm, lumen of crypt; Gb, goblet cells; Pn, paneth cells.

Based on the above histopathological alterations in the colon, we investigated for accompanying alterations in the cytological structure of the crypt of Lieberkuhn in the colon after SPA administration. A TEM analysis of the thin tissue sections of the colon revealed a normal ring structure of the crypts of Lieberkuhn in the CNTR and the SPA-treated groups, in which the enterocytes, goblet cells, and paneth cells are seen to encircle a lumen at the center. However, a dramatic change was observed in the ultrastructure after Lop treatment. An enhancement in the number of lipid droplets was observed in the Lop + Vehicle-treated group, with a significant decrease of the levels or a disappearance of lipid droplets from around the crypt lumen after SPA administration. Moreover, the Lop + SPA-treated group showed an increased number of goblet cells and a decreased number of paneth cells (Figure 4). Taken together, these results indicate that SPA administration effectively induces an increased abundance of lipid droplets and goblet cells in the crypt of the colons in Lop-induced constipated SD rats.

### 2.4. Effect of SPA Administration on Mast Cells Infiltration

**Figure 5:**
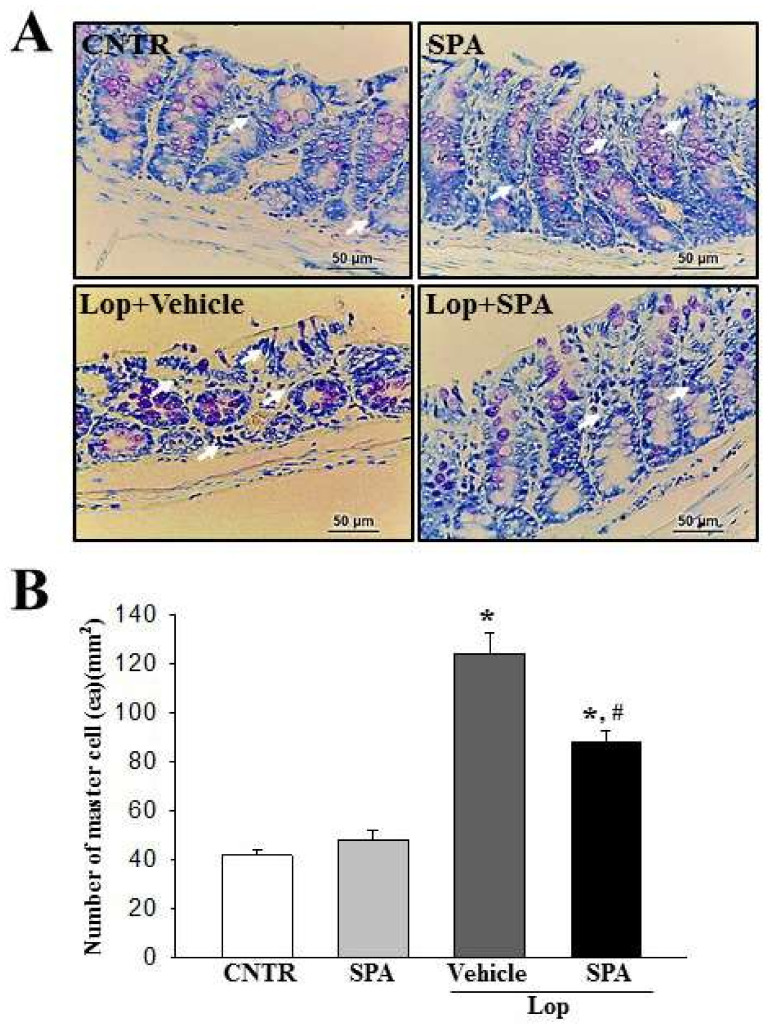
The levels of mast cell infiltration after SPA administration: (**A**) Colon tissue stained with toluidine blue. The infiltration of mast cells (arrows) was identified in the slide sections of the colon tissue by staining with toluidine blue followed by observation at 400× magnification. (**B**) Number of mast cells. Five to six rats per group were used in the toluidine blue analysis, and the cell number was measured in duplicate in three different slides. The data are reported as the mean ± SD. * indicates *p* < 0.05 compared to the CNTR group. # indicates *p* < 0.05 compared to the Lop + Vehicle-treated group.

To examine whether SPA administration can induce the infiltration of mast cells, the number of infiltrated mast cells was measured in the colon of the Lop-induced constipation model. A higher numbers of mast cells stained blue in the Lop + Vehicle-treated group were observed than in the CNTR group. However, their numbers were remarkably decreased by 32% in the Lop + SPA-treated groups (Figure 5A,B). These data suggest that SPA may contribute to the suppression of mast cell infiltration in the colon of the Lop-induced constipation model.

### 2.5. Effect of SPA Administration on the Expression of Inflammatory Cytokines and their Mediators

**Figure 6:**
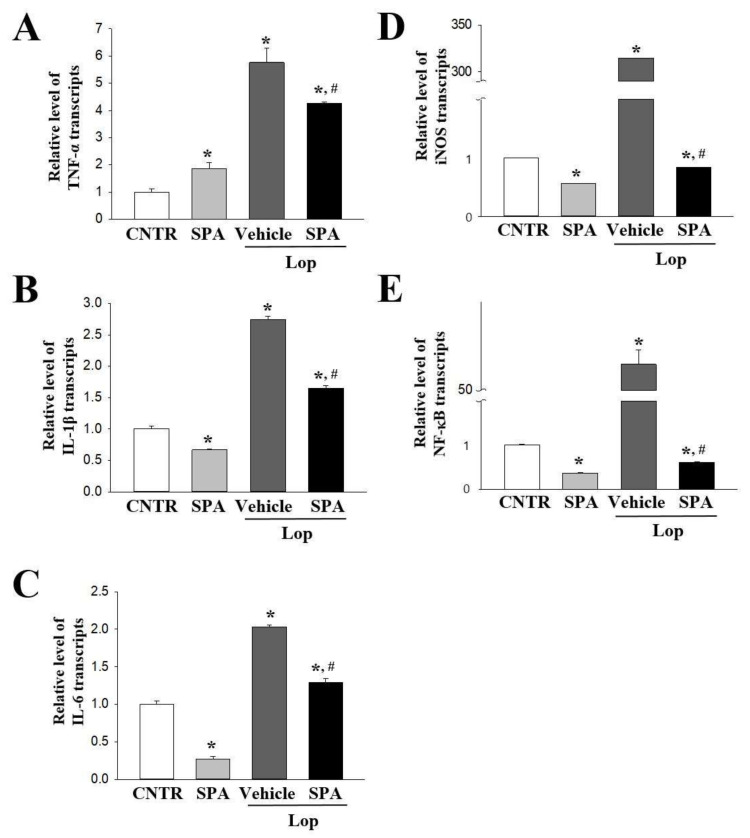
The levels of inflammatory cytokines and their mediators after SPA administration: The transcripts levels of three inflammatory cytokines (TNF-α, IL-1β, and IL-6) (**A**–**C**) and two inflammatory mediators (NK-κB and iNOS) (**D**,**E**) were detected in the colon by a quantitative real-time PCR analysis using specific primers. The intensity of each band was determined densitometrically, and the relative level was calculated based on the intensity of the β-actin transcript as an endogenous control. Four to five rats per group were used in RNA purification, and cytokine levels were assayed in duplicate in each sample. The data are reported as the mean ± SD. * indicates *p* < 0.05 compared to the CNTR group. ^#^ indicates *p* < 0.05 compared to the Lop + Vehicle-treated group.

The expression levels of the proinflammatory cytokines including TNFα, IL-1β, and IL-6 as well as their mediators such as NF-κB and iNOS were measured in the colon using quantitative real-time PCR analysis to evaluate the differences in the inflammatory response among the groups. The transcript levels of three cytokines were increased in the Lop + Vehicle-treated group compared with the CNTR group. However, these levels were significantly decreased in the Lop + SPA-treated group compared with the Lop + Vehicle-treated group (Figure 6). Also, a similar regulation pattern was measured on the inflammatory mediators. The expression level of NF-κB and iNOS were dramatically higher in the Lop + Vehicle-treated group than the CNTR group. However, their levels were remarkably decreased compared with the levels of the Lop + Vehicle-treated groups (Figure 6). These data suggest that SPA administration can suppress the expression of proinflammatory cytokines and their mediators in the colon of the Lop-induced constipation model.

### 2.6. Effect of SPA Administration on Regulation of Inflammation-Derived Mucin Secretion

**Figure 7:**
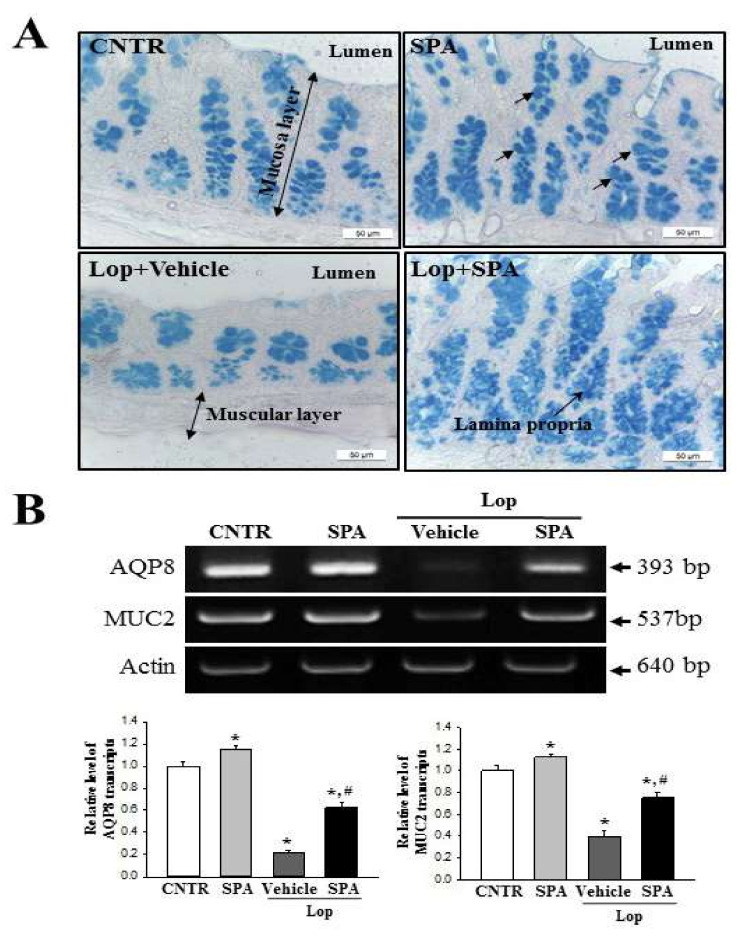
The detection of mucin secretion and membrane water channel expression in the colon after SPA treatment: (**A**) Mucin secreted from the crypt layer cells was stained with Alcian Blue at pH 2.5, and the images were observed at 100× magnification. Five to six rats per group were used in the preparation of the tissue slide and mucin secretion observed in duplicate in three stained slides. (**B**) The levels of MUC2 and AQP8 transcripts in the total mRNA of the colons were measured by RT-PCR using specific primers. After the intensity of each band was determined using an imaging densitometer, the relative levels of MUC2 and AQP8 mRNA were calculated based on the intensity of actin as an endogenous control. Four to five rats per group were used in RNA purification, and each transcript levels were assayed in duplicate in each sample. The data are reported as the mean ± SD. * indicates *p* < 0.05 compared to the CNTR group. ^#^ indicates *p* < 0.05 compared to the Lop + Vehicle-treated group.

Next, to determine if SPA administration regulates the secretion of inflammation-derived mucin in the colon, we evaluated the levels of mucin secretion and the water channel gene transcription in the colon of SD rats. We observed that regions secreting mucin (stained dark blue) were concentrated in the crypts of the mucosa layer of the colon in the CNTR and SPA-treated groups. Lower levels of mucin were observed in the Lop + Vehicle-treated group as compared to the CNTR and SPA-treated groups; however, the levels dramatically increased in the Lop + SPA-treated group (Figure 7A). A similar regulation was observed at the level of the MUC2 transcript. The decrease in the mRNA level of this gene in the Lop + Vehicle-treated group was significantly increased by 2.16-fold in the Lop + SPA-treated group (Figure 7B). Furthermore, we measured the level of AQP8 mRNA to investigate whether mucin secretion induced by SPA treatment is accompanied by an altered expression of the membrane water channel. We detected a similar pattern in the expression level of AQP8 mRNA. Specifically, this level decreased by 62% in the Lop + Vehicle group relative to the CNTR group but dramatically increased 2-fold in the Lop + SPA-treated group (Figure 7B). These results indicate that SPA administration enhances the ability to secrete inflammation-derived mucin and induces the expression of a membrane water channel in the colon of Lop-induced constipated SD rats.

### 2.7. Mechanism of Action of SPA on the Cholinergic Regulation of Gastrointestinal Mobility

**Figure 8:**
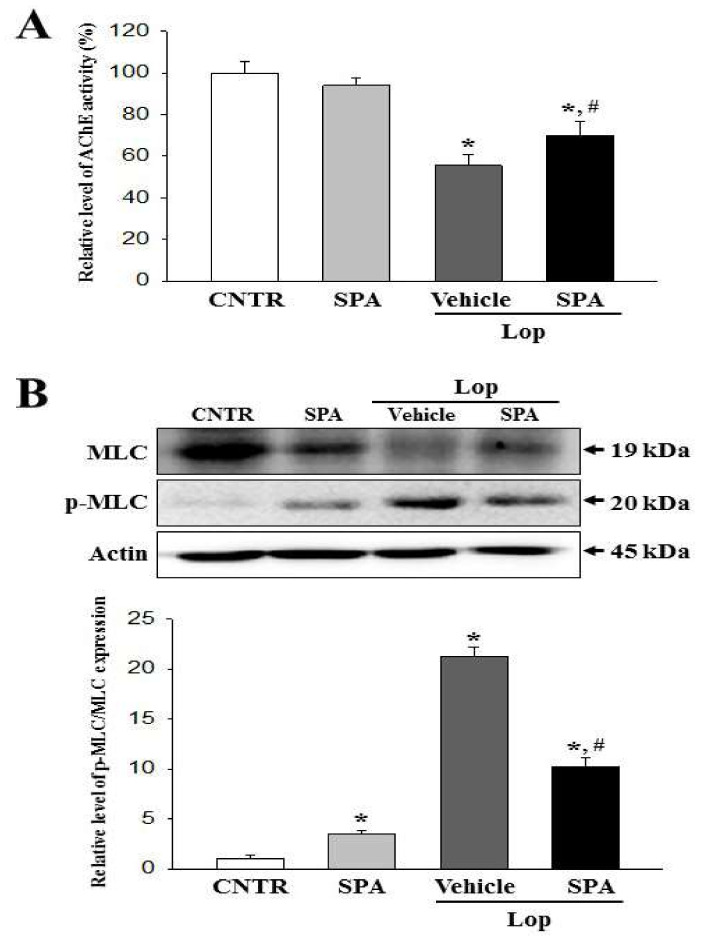
The AChE activity and myosin light chains (MLC) phosphorylation after SPA treatment: (**A**) The measurement of AChE activity. After the homogenization of the colon tissue, the AChE activity was measured using an Acetylcholinesterase Assay Kit. It can detect as little as 0.01 mU AChE in a 100 µl assay volume (0.1 mU/mL). Five to six rats per group were used in preparation of the tissue lysate and AChE activity analysis were assayed in duplicate in each sample. (**B**) The detection of MLC phosphorylation: Expressions of MLC and p-MLC measured by western blot analyses using HRP-labeled secondary anti-rabbit IgG antibody and the relative levels of each protein were calculated relative to the intensity of actin bands. Five to six rats per group were used in the preparation of the protein homogenate and the western blot analysis were assayed in duplicate in each sample. The data are reported as the mean ± SD. * indicates *p* < 0.05 compared to the CNTR group. ^#^ indicates *p* < 0.05 compared to the Lop + Vehicle-treated group.

To investigate the mechanism of action of SPA on the cholinergic regulation for gastrointestinal mobility, we assessed the AChE activity by measuring the AChR M3 downstream signaling pathway in the airway tissue of the Lop-induced constipation model. Our results showed that after the treatment of Lop + Vehicle, the level of AChE activity was lower than in the CNTR group. However, significant increases in the levels were observed in the Lop + SPA-treated groups (Figure 8A). The present results indicate that SPA exposure recovers the secretory ability of ACh in neuronal and nonneuronal cells of the colon to regulate the gastrointestinal mobility.

A recovery pattern was also observed in the AChE activity when assessing the response of the colon smooth muscle cells as target cells for ACh, although they showed an opposite pattern. The phosphorylation level of myosin light chains (MLC) was higher in the Lop + Vehicle-treated group than in the CNTR group. However, these levels were remarkably recovered in the Lop + SPA-treated group (Figure 8B). Taken together, the above results suggest that the SPA-induced cholinergic recovery may be linked to regulating the phosphorylation of MLC in smooth muscle cells.

### 2.8. Molecular Mechanism of the Laxative Effects of SPA on the Downstream Signaling Pathway of mAChRs

**Figure 9:**
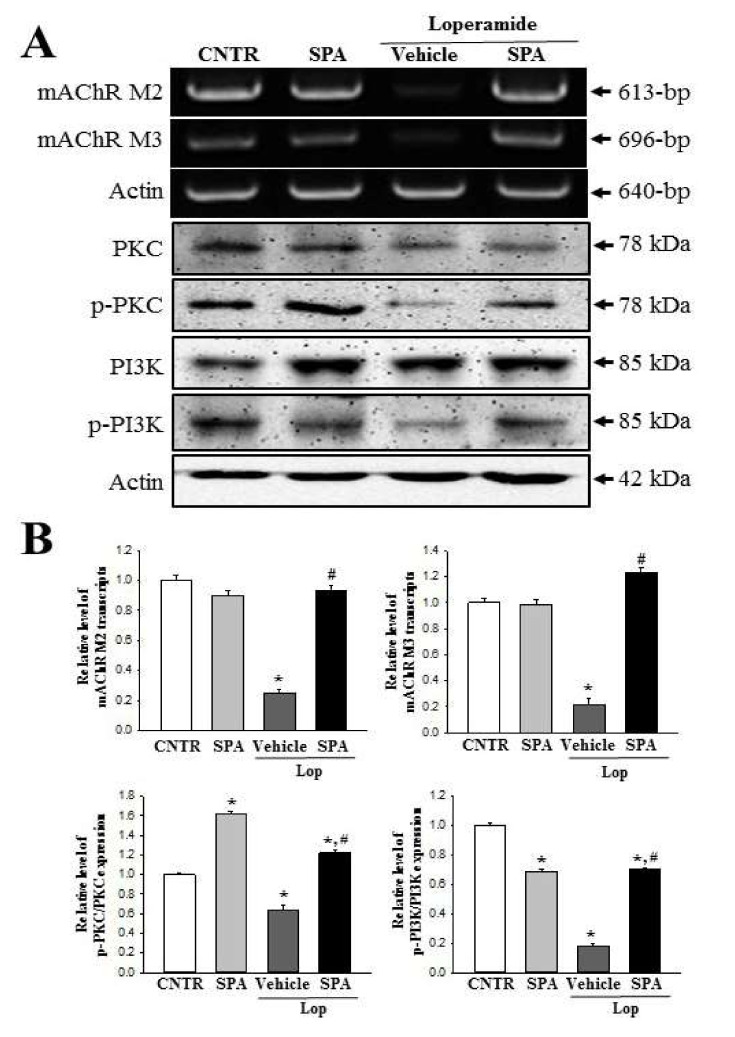
The expression of mAChRs and key mediators within their downstream signaling pathway in the colon after SPA treatment: (**A**) Resulting image of RT-PCR and western blot analyses. The levels of mAChR M2 and M3 transcripts in the total mRNA of colons were measured by semiquantitative PCR using specific primers. After the intensity of each band was determined using an imaging densitometer, the relative levels of mAChR M2 and M3 were calculated based on the intensity of actin. The expression of several related proteins, including PKC, p-PKC, PI3K, and p-PI3K, in the mAChR M2 and M3 signaling pathway were measured by a Western blot analysis using the HRP-labeled anti-rabbit IgG antibody. (**B**) Relative level of each protein expression. After the intensity of each band was determined using an imaging densitometer, the relative levels of the four proteins were calculated based on the intensity of actin. Five to six rats per group were used in the preparation of the protein and RNA, and the western blot and RT-PCR analyses were assayed in duplicate in each sample. The data are reported as the mean ± SD. * indicates *p* < 0.05 compared to the CNTR group. ^#^ indicates *p* < 0.05 compared to the Lop + Vehicle-treated group.

To investigate the correlation between the laxative effects of SPA and the downstream signaling pathway of mAChRs, specific primers and antibodies were applied to evaluate the alterations in the mAChRs expression and key mediators within their downstream signaling pathway in the colon of the Lop + SPA-treated group. The subset group showed similar alterations in the mRNA expression levels of mAChRs M2 and M3. The levels of both receptors were lower in the Lop + Vehicle-treated group than the CNTR group and the SPA-treated group. However, a rapid increase in expression (3.03- and 3.95-fold, respectively) was observed in the Lop + SPA-treated group (Figure 9). Moreover, similar alterations were observed in the expression of key mediators of the mAChR downstream signaling pathway. The overall change in all the experimental groups was similar for the alterations in the expression of PKC, p-PKC, PI3K, and p-PI3K proteins. The phosphorylation levels of these proteins were dramatically recovered in the Lop + SPA-treated group relative to the Lop + Vehicle-treated group, although the Lop + Vehicle-treated group exhibited lower or higher levels than the CNTR and SPA groups (Figure 9). Taken together, the results of this study suggest that the laxative effects induced by SPA treatment correlate with the recovery of the downregulation of the mAChRs expression and their downstream signal in the colons of constipated SD rats.

## 3. Discussion

Attention has recently been focused on several herbal plants and natural products as novel therapeutic drugs for the treatment of gastrointestinal diseases due to their significant potential to alleviate symptoms of such diseases [5,7,9]. In an effort to identify novel drugs for the treatment of constipation, we investigated the inflammatory responses and the cholinergic regulation exerted during the laxative effects of SPA in Lop-induced constipated SD rats. *A. cochinchinensis* roots have been implicated in the recovery of neuronal cells and gut injury in studies using animals and Drosophila [22,23,24]. The results of the current study clearly demonstrate that SPA induces laxative effects, including the elevation of stool excretion and the recovery of histological changes induced by Lop injection in the colon. Furthermore, we believe our data to be the first to demonstrate that SPA suppresses proinflammatory cytokines and their mediators, upregulates mucin secretion, as well as downregulates the mAChRs signaling pathway while effectively alleviating constipation.

The main constituents of the tuberous roots of *A. cochinchinesis* consist of 19 amino acids, polysaccharides, and more than 20 multifunctional compounds [25,26,27]. These functional compounds include β-sitosterol [3], daucosterol [4], n-ethatriacontanoic acid [5], palmitic acid [9], 9-heptacosylene [10], smilagenin [11], diosgenin [12], sarsasapogenin-3-O-β-D-glucoside feeding grapes imidacloprid [13], 5-methoxy methyl furfural, yame sapogenin, diosgenin-3-O-β-D imidacloprid feeding glucose glycosides [13,14], aspacochioside D [7], iso-agatharesinoside [15], and seven steroidal saponins [21]. The roots of *A. cochinchinesis* have several therapeutic properties, including antiaging and antioxidant effects [22,28,29], antibacterial inflammatory effects [22,26,27], anticancer effects [23,24,30], and hepatoprotective activity [25]. Moreover, they are administered in combination with other herbs to treat lung diseases, immune system-related diseases, and aging [9,10]. Recently, several studies for the medicinal use with *A. cochinchinensis* suggest its therapeutic effects for constipation, since constipation is tightly associated with the dysfunction of the neuronal and digestive systems. The root extract with *A. cochinchinensis* significantly increases the activity of superoxide dismutase (SOD) and reduces the contents of malondialdehyde (MDA) in the brain [23]. Moreover, an aqueous extract with *A. cochinchinesis* stimulates the nerve growth factor (NGF) expression and secretion in neuronal cells [24]. Furthermore, changes associated with gut injuries induced by metal ion treatment, such as the survival rate of epithelial cells and morphological changes in the gut, were effectively alleviated by treatment with the aqueous *A. cochinchinesis* extract [25]. However, the laxative effects of *A. cochinchinesis* have not been examined in a constipated animal model to date, although the laxative effects of *L. platyphylla* are well-studied. This present study provides strong evidence of the novel function of *A. cochinchinesis* extract containing high concentrations of saponin, although further studies are required to understand how saponin affects the suppression of the inflammatory response and regulates the motility of the gastrointestinal tract.

Some extracts and compounds derived from *A. cochinchinensis* roots are reported to induce anti-inflammatory effects in various diseases. The ethanolic extract from *A. cochinchinensis* and methyl protodioscin (MP) effectively reduce acute and chronic skin inflammation induced by 12-O-tetradecanoyl-phorbol-13-acetate (TPA)-induced mouse ear edema and inhibits the airway inflammation in a lipopolysaccharide (LPS)-induced acute lung injury model [31,32]. A SPA and butanol extract of *A. cochinchinensis* roots fermented with *Weissella cibaria* (BAW) have been shown to prevent airway inflammation and remodeling in the lung tissue of an ovalbumin (OVA)-induced asthma model [33,34]. However, there have been no studies investigating the anti-inflammatory response of *A. cochinchinensis* roots in the gastrointestinal tract of animals with inflammatory disease in spite of the above researches providing few evidences. Our results, therefore, present a clue for the novel role of SPA in alleviating the symptoms of constipation accompanied by an inflammatory response in the colon of the Lop-induced model. Furthermore, the ameliorating effects of SPA on the inflammatory mechanism is very similar to the outcomes observed in the atractylodin-treated constipation-prominent model [6]. Especially, the increased levels of proinflammatory cytokines were remarkably reversed after exposure to SPA and atractylodin, although the decrease rate was greater in the SPA-treated group than in atractylodin-treated group. Moreover, the results of the present study provide clues to the advantages or disadvantages for a single treatment of SPA in the physiological condition of SD rats. This extract promoted the decrease of paneth cells number and some cytokines expression as well as the enhancement of urine volume. At the same time, SPA induced the increase of PKC phosphorylation and the decrease of PI3K phosphorylation. These properties of SPA were showed the possibility that SPA can be considered as a versatile substance to treat human diseases.

Few studies have provided a theoretical basis for microbial treatment in chronic constipation by demonstrating a connection between constipation and intestinal flora disturbance. One treatment includes probiotics, prebiotics, symbiotics, and fecal microbiota transplantation (FMT) and is widely accepted because of the safety, convenience, and curative nature [35]. The oral administration of *Lactobacillus casei* Qian for 9 days induces an increase in the gastrointestinal transit, feces number, and some gastrointestinal hormone levels in carbon-induced constipated mice [36]. In patients with gastrointestinal symptoms, treatment with EpiCor^®^ fermentate (consisting of dried yeast extracts) for 2 weeks improves the constipation-related symptoms by modulating the gut microbiome [37]. Most studies evaluating the therapeutic effects of microbial treatment require administration and observation for a duration exceeding 1 week. However, in the current study, SPA was administered only once and the observation was for 2 days. Hence, we were unable to consider the laxative effects induced by various metabolites of SPA on the disturbance of gut microbiota during our study; future long-term studies are required to confirm whether fermentable saccharides affect the excretion of stool. Meanwhile, some components such as saponin, flavonoids, and polyphenol included in SPA can modulate the composition of gut microbiota through the suppression of pathogen growth and the promotion of beneficial bacteria growth [38]. These alterations of intestinal microbiota regulated gut sensory and motor functions and then improved chronic constipation. Therefore, the regulation of gut microbiota was considered as one of the new therapeutic tools for the management of chronic constipation [39]. Also, microbial metabolites short-chain fatty acid (SCFAs) mainly produced in the colon by the bacterial fermentation of carbohydrate can be considered as another regulating factor for laxative effects because SCFAs stimulate the increase of transit time [40,41].

A chemically synthesized drug, Lop decreases the risk of dehydration and systemic or metabolic diseases such as diarrhea. Due to these properties, it is widely applied to induce constipation by administering 1.5–3 mg/kg body weight of Lop for 3–7 days in laboratory animals [35,36,37,42,43]. Lop stimulates the extension of the stool evacuation time and the delay of intestinal luminal transit by inhibiting water secretion [44] and smooth muscle movement of the intestinal wall [45,46]. However, the efficacy of Lop differs depending on the specific target of their mechanism of action. Diarrhea was controlled for 2 or 8 h following the oral administration of Lop in the castor oil diarrhea test using rats [47]. In patients with irritable bowel syndrome treated with 4–12 mg of Lop, the prolongation of the whole gut transit lasted from 42 to 56 h [48]. In our study, an alteration of the laxative parameters, including stool excretion, histological structure, signaling pathway of mAChR and mucin secretion, were evaluated in Vehicle- or SPA-treated SD rats at 48 h after the final Lop treatment. The constipation induced by Lop was consistently maintained for 48 h in the Lop + Vehicle-treated group, while the Lop + SPA-treated group showed significant laxative effects. Hence, we believe that the constipation effect of Lop administration on the histological structure of the colon can be completely excluded in SD rats at 48 h after SPA administration. 

Food intake and water consumption are important factors to be considered when investigating the process leading to symptoms of constipation and the subsequent therapeutic effects of laxative drugs. In the constipation model, several herbal medicines and foods induce a variety of responses with regards to food intake and water consumption. Aqueous leaf extract of *A. ferox* Mill. induces enhanced water consumption without any significant alteration of food intake [12], while the reverse was observed in a constipation model treated with an aqueous extract of *L. platyphylla* (AEtLP) [5]. Moreover, food intake and water consumption were significantly decreased in the constipation model treated with a *Ficus carica* paste [15]. However, a previous study of GEGR [7] and the current investigation of SPA reveal no significant alterations in food intake or water consumption. These differences might be associated to unknown factors such as the innate taste of the herbal medicines and foods used in each study.

In most constipation studies, a key marker of constipation is the significant reduction in stool excretion in the Lop-induced animals. Previously, stool-related factors including stool number, weight, and water content dramatically decreased in the Lop-induced constipation model [5,7,10,12,22]. However, several plant extracts and foods with laxative effects induce the recovery of these alterations. Leaf extracts of *Mareya micrantha* and *A. ferox* Mill., a widely used medicinal plant with healing properties, are known to improve the number, water content, and weight of stools in Lop-induced rats [10,12]. Furthermore, relative to a control group, AEtLP and GEGR increase the stool output of rats exposed to 1000 mg/kg or 250–1000 mg/kg, respectively [5,7]. In the current study, a similar effect on stool-related factors was observed in the Lop-induced constipation model treated with 1000 mg/kg of SPA, although some differences were observed in the concentration of the treated extracts.

Several significant alterations in histological and cytological structures are detected in the colons of animal models with constipation. The thickness of the colon layer significantly decreases after Lop treatment [5], and the average thickness of the mucus layer, muscle, and flat luminal surface is thinner in the Lop-treated groups than the vehicle-treated groups [7]. However, these alterations recovered completely after treatment with several natural products, including AEtLP and GEGR [5,7]. As presented in Figure 3, the histological alterations of the colons observed in the present study were very similar to those of previous studies reported by Kim et al. [5,7]. Especially, the flat luminal surface including the outer mucus layer of the colon acts as the first defense line to protect the epithelium from bacteria, inhibiting inflammation and infection [49]. Their thickness can be altered under various pathological conditions such as ulcerative colitis, Crohn’s disease, and cancer [50]. Similar patterns were observed in the colon of Lop-induced constipated SD rats. Furthermore, an analysis of the cytological structures revealed similar responses in the crypt of Lieberkühn, including enterocytes, goblet cells, and Paneth cells [5,7]. Lipid droplets containing mucin accumulate in the cytoplasm of goblet cells and enterocytes following the induction of constipation, whereas abundant granules were present in paneth cells. However, Lop + SPA administration stimulated the secretion of lipid droplets into the crypt lumen in both goblet cells and enterocytes. These alterations were similarly detected in the Lop-induced constipation model treated with AEtLP and GEGR [5,7]. Therefore, our results provide additional evidence that ultrastructural changes in all three types of crypt cells are tightly correlated with the progression of Lop-induced constipation and the recovery with laxative drugs. However, some additional studies including Ussing-like chamber analysis are needed to justify the recovery of mucus thickness in the colon of a Lop-induced constipation model after SPA treatment.

Finally, based on two previous studies, we selected mAChRs as the target protein for the investigation of the molecular mechanism responsible for the laxative effects of SPA [5,7]. The results of the previous studies report significant alterations in the levels of the mAChR M2 transcripts in the colon after Lop administration. The levels of mAChR M2 and M3 transcripts dramatically increased in the colon of SD rats during Lop-induced constipation; however, these increases decreased after AEtLP and GEGR treatment. Furthermore, the expression patterns of PI-3K and PKC in the downstream signaling pathway were reported to be very similar to that of mAChR M2 and M3. Also, the increase in PI-3K and PKC expression induced by Lop administration was inhibited by AEtLP and GEGR treatment [5,7]. In the current study, we observed similar alterations in the mAChR M2 and M3 signaling pathway in the Lop + SPA-treated group, although there were some variations in their range of alteration. We postulate that the alterations in the mAChR M2 and M3 signaling pathway observed in the present study can provide information for use in future investigations to assess the causes of constipation and to help in the selection of targets for treating constipation-related diseases.

## 4. Materials and Methods

### 4.1. Preparation of SPA

We obtained fresh roots of *A. cochinchinensis* from the Gochang National Agricultural Cooperation Federation in Korea. After washing, the roots obtained were dried in a drying machine (FD5510S-FD5520S, Ilshinbiobase Co., Seoul, Korea) at 60 °C. Voucher specimens of *A. cochinchinensis* roots (WPC-14-003) were deposited in the functional materials bank of the Wellbeing RIS Center at Pusan National University. The samples were also identified by Shin Woo Cha at the Herbal Crop Research Division, National Institute of Horticultural & Herbal Science. The dried *A. cochinchinensis* roots were then reduced to a powder using a pulverizer (MF-3100S, Hanil Electric Co., Seoul, Korea), after which SPA was obtained via extraction for 24 h at 50 °C using a fixed liquor ratio (ratio of solid powdered *A. cochinchinensis* to ethyl acetate solvent, 1:10) in a circulating extraction equipment (SHWB-30/45; Woori Science Instrument Co., Pocheon, South Korea). The SPA was then concentrated and subsequently passed through a 0.4 µm filter, after which the pellets were dried in a rotary evaporator (EYELA, Tokyo, Japan) and stored at −80 °C until used. The collected SPA powder was finally dissolved in distilled water (in dH_2_O) to 600 mg/kg and diluted further to the required concentration before use.

Meanwhile, SPA contained polyphenols (102.1 μg/g), total flavonoid (88.5 μg/g), and crude saponins (55.7 mg/g). The IC_50_ value of SPA on the inhibitory activity of the DPPH radical was determined at 587.6 μg/mL [33].

### 4.2. Experimental Design for Animal Study

The Pusan National University-Institutional Animal Care and Use Committee (PNU-IACUC) reviewed and approved the animal protocol for the therapeutic effects of laxative drug based on the ethical procedures for scientific care (Approval Number PNU-2015-0952). The Pusan National University-Laboratory Animal Resources Center, accredited by the Korea Food and Drug Administration (KFDA)(Accredited Unit Number-000231) and The Association for Assessment and Accreditation of Laboratory Animal Care (AAALAC) International (Accredited Unit Number; 001525), handled the adult Sprague Dawley (SD) rats, which were purchased from Samtako BioKorea Inc. (Osan, Korea). The animals were provided with access to a standard irradiated chow diet (Samtako BioKorea Inc.) and water *ad libitum*. Additionally, rats were maintained in a specific pathogen-free (SPF) state under a strict light cycle (on at 08:00 h; off at 20:00 h) at 23 ± 2 °C and 50 ± 10% relative humidity throughout the experiment.

The induction of constipation, the administration schedule of the laxative drug, and the measurement method of several related parameters were performed as per protocols described previously [7,14]. The optimal dose of SPA was determined based on our preliminary data which revealed a dose-dependent increase in stool excretion after exposures to 250, 500, and 1000 mg/kg of SPA in SD rats. We selected 1000 mg/kg of SPA as the optimal dosage for the laxative effect because it showed maximal effects without any specific toxicity. In the current study, 8-week-old SD rats (*n* = 28) were assigned to either a non-constipation group (CNTR group, *n* = 14) or a constipation group (*n* = 14). A subcutaneous injection of Lop (4 mg/kg weight) in 0.5% Tween 20 in saline was administered twice daily to induce constipation (9 a.m. and 6 p.m.) for 3 days, whereas the non-constipation group received 0.5% Tween 20 in saline alone under the same pattern. The non-constipation group was further divided into a CNTR group (*n* = 7) and a SPA-treated group (*n* = 7). At 9 a.m. on the 4th day, the CNTR group did not receive any solution, whereas the SPA-treated group was orally administered a single dose of 1000 mg/kg of SPA. Additionally, the constipation group was divided into a Lop + Vehicle-treated group (*n* = 7) and a Lop + SPA-treated group (*n* = 7). The Lop + SPA-treated groups were orally administered a single dose of 1000 mg/kg body weight SPA, while the Lop + Vehicle-treated group received the same volume of 1× phosphate buffer saline (PBS) under the same pattern. At 9 a.m. on the 5th day, the total stools, urine, water, and food were collected from the metabolic cage of each group, and the levels were measured using appropriate methods. Moreover, all SD rats were euthanized using CO_2_ gas, after which the tissue samples were acquired and stored at −70 °C in Eppendorf tubes until assay. 

### 4.3. Gastrointestinal Transit Ratio Analysis

The gastrointestinal motility was measured by the method described by Choi et al. [51] and Kim et al. [52] with some modifications. Briefly, SD rats were fasted for 18 h prior to the experiment but were allowed to consume water *ad libitum*. These rats were fed with 1 mL of charcoal meal (3% suspension of activated charcoal in 0.5% aqueous methylcellulose) (Sigma-Aldrich Co., St. Louis, MO, USA). After 30 min of treatment, these rats were euthanized using CO_2_ and the intestinal tract was collected from the abdominal cavity. The intestinal charcoal transit ratio was calculated as follows:

Charcoal transit ratio (%) = [(Total intestine length − transit distance of charcoal meal)/total intestine length] × 100(1)

### 4.4. Analysis of Food Intake, Water Intake, and Body Weight

The food weight, water volume, and body weight of SD rats treated with Vehicle or SPA were measured at 9 a.m. on the 5th day using an electrical balance (for food and body weight) and a measuring cylinder (for water volume). The average food intake and water consumption were then calculated using the above data. All measurements were performed three times to ensure the accuracy.

### 4.5. Measurement of Stool Parameters and Urine Volume

SD rats were individually bred in metabolic cages throughout the experiment to provide uncontaminated stool and urine samples (Daejong Instrument Industry Co., LTD, Seoul, Korea). The stool number and weight were measured as previously described [5,12]. Briefly, stools excreted from each SD rat were collected at 9 a.m. from the 1st day to the 5th day, weighed three times each using an electric balance, and counted three times. The stool moisture content was also analyzed as follows:

Stool moisture content = (A − B)/A × 100(2)

where A is the weight of fresh stools collected after Lop administration, and B is the weight of stools after drying at 60 °C for 12 h. Furthermore, the urine volume collected at 9 a.m. on the 5th day was measured three times per sample using a cylinder.

### 4.6. Western Blotting

Pro-Prep Protein Extraction Solution (Intron Biotechnology Inc., Seongnam, Korea) was used to obtain the total proteins from the colons of subset groups (CNTR, SPA-, Lop + Vehicle-, and Lop + SPA-treated SD rats). The acquired proteins were subsequently centrifuged at 13,000 rpm and 4 °C for 5 min, after which the protein concentrations were determined using a SMARTTM Bicinchoninic Acid Protein assay kit (Thermo Fisher Scientific Inc.). The proteins (30 μg) were subjected to 4%–20% sodium dodecyl sulfate-polyacrylamide gel electrophoresis (SDS-PAGE) for 3 h, and the resolved proteins were transferred to nitrocellulose membranes for 2 h at 40 V. The membranes were then probed with the following primary antibodies, overnight at 4 °C: anti-PI-3K (Cell Signaling Technology Inc., Cambridge, MA, USA), anti-p-PI3K (Cell Signaling Technology Inc.), anti-PKC (Cell Signaling Technology Inc.), anti-p-PKC (Cell Signaling Technology Inc.), anti-MLC (1:1000, Abcam, Cambridge, UK), anti-p-MLC (1:1000, Abcam), or anti-actin (Sigma-Aldrich Co.). After washing with a washing buffer (137 mM NaCl, 2.7 mM KCl, 10 mM Na_2_HPO_4_, 2 mM KH_2_PO_4_, and 0.05% Tween 20), the membranes were incubated with 1:1000 diluted horseradish peroxidase-conjugated goat anti-rabbit IgG (Zymed Laboratories, South San Francisco, CA, USA) for 2 h at room temperature, after which the blots were developed using a Chemiluminescence Reagent Plus kit (Pfizer Inc., Gladstone, NJ, USA). Signal images of each protein were subsequently acquired using a digital camera (1.92 MP resolution) of the FluorChem^®^ FC2 Imaging system (Alpha Innotech Corporation, San Leandro, CA, USA). The protein densities were semi-quantified using the AlphaView Program version 3.2.2 (Cell Biosciences Inc., Santa Clara, CA, USA).

### 4.7. Semiquantitative PCR Anlysis

The total RNA was isolated from the frozen tissue of the colons using RNAzol B solution (Tet-Test Inc.) according to the manufacturer’s protocols. Following the synthesis of cDNA, the genes were amplified by subjecting the samples to 28 cycles of 30 s at 94 °C, 30 s at 62 °C, and 45 s at 72 °C in a Perkin-Elmer Thermal Cycler. The primer sequences used to evaluate various gene expressions were as follows: mAChR M2 sense primer 5′-CCAGT ATCTC CAAGT CTGGT GCAAG G-3′ and antisense primer 5′-GTTCT TGTAA CACAT GAGGA GGTGC-3′; mAChR M3 sense primer 5′-GTCAC TTCTG GTTCA CCACC AAGAG C-3′ and antisense primer 5′-GTGTT CACCA GGACC ATGAT GTTGT AGG-3′; AQP8 sense primer 5′-GTAGT ATGGA CCTAC GTGAG ATCAA GG-3′ and antisense primer 5′-AGAAC CTTTC CTCTG GACTC ACCAC C-3′; MUC2 sense primer 5′-GCTGC TCATT GAGAA GAACG ATGC-3′ and antisense primer 5′-CTCTC CAGGT ACACC ATGTT ACCAG G-3′; and β-actin sense and antisense primers 5′-TGGAA TCCTG TGGCA TCCAT GAAAC-3′ and 5′-TAAAA CGCAG CTCAG TAACA GTCCG-3′, respectively. All PCR products were quantified using 1% agarose gels and a Kodak Electrophoresis Documentation and Analysis System 120.

### 4.8. Quantitative Real-Time PCR Analysis

Quantitative real-time PCR assessed the relative quantities of mRNA for inflammatory cytokines. The total RNA molecules were isolated from frozen colon tissues using RNA Bee solution (Tet-Test Inc., Friendswood, TX, USA). After the quantification of the RNA concentration, the complement DNA (cDNA) was synthesized using a mixture of oligo-dT primer (Invitrogen, Carlsbad, CA, USA), dNTP, and reverse transcriptase (Superscript II, 18064-014, Invitrogen; Thermo Fisher Scientific, Inc., Waltham, MA, USA). Q-PCR was then conducted using a cDNA template and 2×Power SYBR Green (TOYOBO Co., Osaka, Japan) as described in previous studies [34]. The primer sequences used to evaluate the various gene expressions were as follows: TNF-α sense primer 5′-CCTGT AGCCC ACGTC GTAGC-3′ and antisense primer 5‘-TTGAC CTCAG CGCTG ACTTG-3′; IL-1β sense primer 5′-GCACA TCAAC AAGAG CTTCA GGCAG-3′ and antisense primer 5′-GCTGC TTGTG AGGTG CTGAT GTAC-3′; IL-6 sense primer 5′-TTGGG ACTGA TGTTG TTGACA-3′ and antisense primer 5′-TCATC GCTGT TGATA CAATC AGA-3′; and β-actin sense and antisense primers 5′-TGGAA TCCTG TGGCA TCCAT GAAAC-3′ and 5′-TAAAA CGCAG CTCAG TAACA GTCCG-3′, respectively. The reaction cycle at which PCR products exceeded this fluorescence intensity threshold during the exponential phase of PCR amplification was considered as the threshold cycle (CT).

### 4.9. Histopathological Analysis

The colons collected from CNTR, SPA-, Lop + Vehicle-, and Lop + SPA-treated SD rats were fixed with 10% formalin for 48 h. The samples were subsequently embedded in paraffin wax, after which they were cut into 4 μm thick sections and stained with hematoxylin and eosin (H&E, Sigma-Aldrich Co.). The sections were then analyzed for mucosa thickness, flat luminal surface thickness, and number of goblet cells in the colons by light microscopy and the Leica Application Suite (Leica Microsystems, Switzerland).

The infiltration of mast cells into the colon was detected by staining with toluidine blue as previously described. After deparaffinization and dehydration, these sections were stained with 0.25% Toluidine blue (Sigma-Aldrich, MO, USA) and measured by light microscopy for the total number of mast cells. The number of cells per specific area was measured using the Leica Application Suite (Leica Microsystems, Wetzlar, Germany).

Mucin staining was conducted by fixing the colons collected from SD rats of all subset groups with 10% formalin for 48 h, then embedding the samples in paraffin wax, and sectioning them into 4 μm thick slices that were subsequently deparaffinized with xylene and rehydrated. The morphological features in the stained colon sections were then observed by light microscopy after the mounted tissue sections were rinsed with distilled water and stained using an Alcian Blue Stain kit (IHC WORLD, Woodstock, MD, USA).

### 4.10. Ultrastructure Analysis Using Transmission Electron Microscopy (TEM)

Colons collected from CNTR, SPA-, Lop + Vehicle-, and Lop + SPA-treated SD rats were fixed in 2.5% glutaraldehyde in a 1× PBS buffer. Next, the samples were washed, after which they were dehydrated with ascending concentrations of ethanol, incubated in 1% OsO_4_ for 1 h at room temperature, and finally embedded in Epon812 media (Polysciences Inc., Eppelheim, Germany). The embedded samples were then cut into ultrathin sections (70 nm) that were subsequently collected on holey formvar coated grids, contrasted by treatment with uranyl acetate and lead citrate, and examined by TEM (Hitachi, Tokyo, Japan) at 4000× magnification.

### 4.11. AChE Activity Analysis

The AChE activity was determined using an Acetylcholinesterase Assay Kit (Abcam, Cambridge, UK) according to the manufacturer’s protocols. Briefly, the colons of each rats was homogenized in a PRO-PREP protein extraction solution (1.0 mM PMSF, 1.0 mM EDTA, 1.0 μM pepstatin, 1.0 μM leupeptin, and 1.0 μM aprotinin) (iNtRON Biotechnology Inc., Seoul, Korea), after which the homogenates were stored at −70 °C until analysis. The sample or standards and the ACh reaction mixture were then incubated on a 96-well plate for 10 min at room temperature while protected from the light. Color alterations were read using a Vmax plate reader (Molecular Devices, Sunnyvale, CA, USA) at 410 nm.

### 4.12. Statistical Analysis

The statistical significance was evaluated using One-way Analysis of Variance (ANOVA) (SPSS for Windows, Release 10.10, Standard Version, Chicago, IL, USA) followed by Tukey post hoc t-test for multiple comparison. All the values were expressed as the means ± SD, and a *p* value < 0.05 was considered statistically significant.

## 5. Conclusions

Taken together, our study results indicate that SPA administration induces the recovery of stool number, water content, urine quantity, and gastrointestinal transit, while enhancing the mucosa thickness, flat luminal surface thickness, and the number of goblet cells in Lop-induced constipation. These results provide evidence that the laxative effects of SPA are accompanied with the suppression of inflammatory responses and the recovery of muscarinic cholinergic regulation. In conclusion, these findings indicate that saponin derived from various herbal plants is a potential therapeutic candidate for the treatment of constipation, although many additional studies are required to confirm this.

## Figures and Tables

**Table 1:** The measurement of body weight, feeding behavior, and urine secretion in loperamide (Lop)-induced constipated Sprague Dawley (SD) rats.

Contents		NCTR	SPA	Lop	
				Vehicle	SPA
Body weight (g)		289 ± 17	288 ± 7	279 ± 11	283 ± 12
Feeding behavior	Food intake (g/day)	18.7 ± 3.6	23.0 ± 1.2	22.0 ± 3.28	23.8 ± 1.8
	Water consumption (ml/day)	29.4 ± 3.4	27.7 ± 3.4	26.1 ± 4.1	25.4 ± 3.6
Urine volume (mL/day)		11.6 ± 5.6	18.3 ± 2.1 *	21.6 ± 2.8 *	16.0 ± 1.6 #

Seven rats per group were used in all contents, and each parameter was assayed in triplicate in each test. The data are reported as the mean ± SD. * *p* < 0.05 compared to the non-constipation (CNTR) group. # *p* < 0.05 compared to the Lop + Vehicle-treated group. SPA; saponin-enriched *A. cochinchinesis* extract.

## Abbreviations

SPA	saponin-enriched extracts of *Asparagus cochinchinensis*
Lop	loperamide
AChE	acetylcholine esterase
MLC	myosin light chain
AEtLP	aqueous extract of *L. platyphylla*
RLP	Red *L. platyphylla*
GEGR	gallotanin-enriched galla rhois
CLP	cecal ligation and puncture
DSS	dextran sulfate sodium
HPLC	high performance liquid chromatography
DPPH	2,2-diphenyl-1-picrylhydrazyl
KFDA	Korea Food and Drug Administration
AAALAC	Association for Assessment and Accreditation of Laboratory Animal Care
SD	Sprague Dawley
SPF	specific pathogen-free
RT-PCR	Reverse transcription polymerase chain reaction
H&E	hematoxylin and eosin
TEM	transmission electron microscopy
SOD	superoxide dismutase
MDA	malondialdehyde
NGF	nerve growth factor
MP	methyl protodioscin
TPA	12-O-tetradecanoyl-phorbol-13-acetate
LPS	Lipopolysaccharide
BAW	butanol extract of *A. cochinchinensis* roots fermented with *Weissella cibaria*
OVA	ovalbumin
FMT	fecal microbiota transplantation
