# Correction: Kim et al. Anti-Inflammatory Response and Muscarinic Cholinergic Regulation During the Laxative Effect of *Asparagus cochinchinensis* in Loperamide-Induced Constipation of SD Rats. *Int. J. Mol. Sci.* 2019, *20*, 946

**DOI:** 10.3390/ijms27052228

**Published:** 2026-02-27

**Authors:** Ji Eun Kim, Ji Won Park, Mi Ju Kang, Hyeon Jun Choi, Su Ji Bae, You Sang Choi, Young Ju Lee, Hee Seob Lee, Jin Tae Hong, Dae Youn Hwang

**Affiliations:** 1 Department of Biomaterial Science, College of Natural Resources and Life Science/Life and Industry Convergence Research Institute, Pusan National University, Miryang 627-706, Republic of Korea; prettyjiunx@naver.com (J.E.K.); pjw08260824@naver.com (J.W.P.); beautifulbead@naver.com (M.J.K.); rudwns546@naver.com (H.J.C.); suji130501@naver.com (S.J.B.); choiyusang@gmail.com (Y.S.C.); youngju0831@naver.com (Y.J.L.); 2 College of Human Ecology, Pusan National University, Busan 609-735, Republic of Korea; heeseoblee@pusan.ac.kr; 3 College of Pharmacy, Chungbuk National University, Chungju 361-763, Republic of Korea; jinthong@chungbuk.ac.kr

In the original publication [1], there was a mistake in Figure 4A as published. A TEM analysis for the ultrastructure of the colon was conducted to investigate the laxative effect of SPA. In the original figure, the “CNTR group” and “Lop + Vehicle group” were wrong. These TEM images were duplicated with those of the No group and Vehicle group in our published papers of another journal due to the negligence of insufficient careful inspection. In addition, the authors once again confirmed the accuracy of these figures from raw data for TEM images. The corrected version of Figure 4A appears below. The authors state that the scientific conclusions are unaffected. This correction was approved by the Academic Editor. The original publication has also been updated.

## Figures and Tables

**Figure 4:**
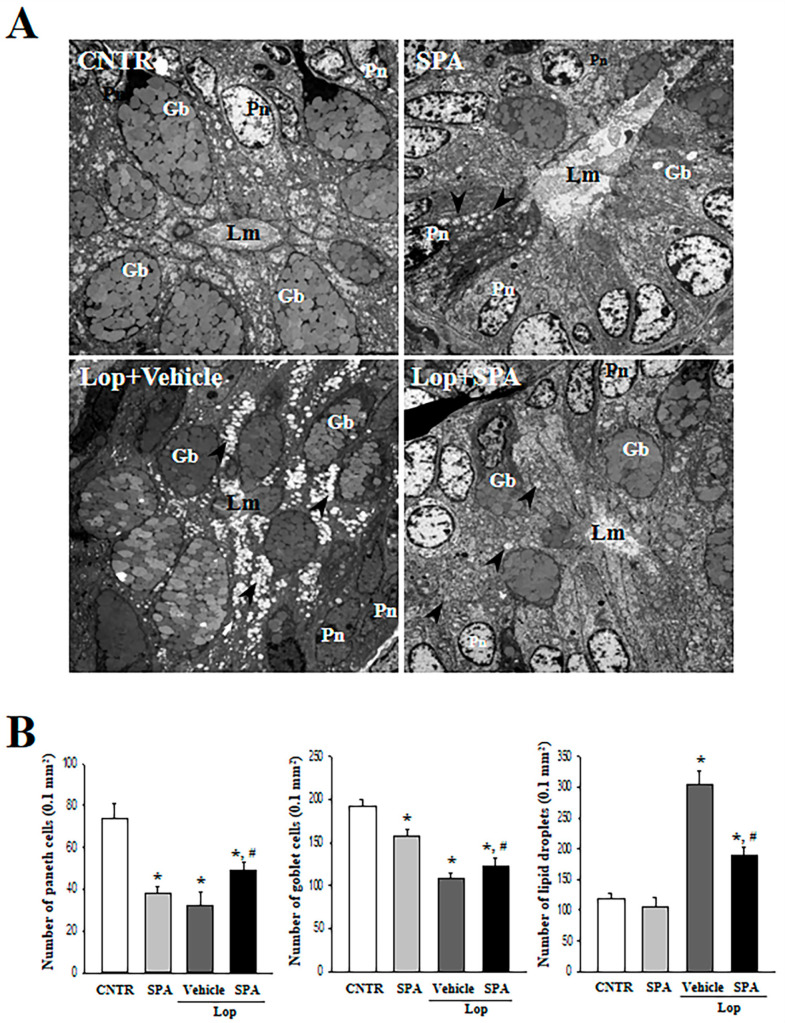
An ultrastructure image of the colon after SPA administration: (**A**) The ultrastructure of the crypt in the CNTR, SPA-, Lop + Vehicle- and Lop + SPA-treated groups were viewed by TEM at 4000× magnification. (**B**) The number of paneth cells, lipid droplets, and goblet cells were measured using Leica Application Suite (Leica Microsystems, Switzerland). The arrow indicates a lipid droplet distributed around the lumen of the crypt. Two to three rats per group were used in the TEM analysis, and each parameter was measured in duplicate in two different slides. The data are reported as the mean ± SD. * indicates *p* < 0.05 compared to the CNTR group. # indicates *p* < 0.05 compared to the Lop + Vehicle-treated group. Lm, lumen of crypt; Gb, goblet cells; Pn, paneth cells.
